# A Scottish Treasurer's View of the Outlook

**Published:** 1912-01-06

**Authors:** J. D. Hedderwick

**Affiliations:** Glasgow Royal Infirmary, Glasgow


### A SCOTTISH TREASURER'S VIEW OF THE OUTLOOK.

MR. J. D. HEDDERWICK, Chairman of the Trustees and Treasurer Glasgow Royal Infirmary.

*To the Editor of* THE HOSPITAL.

SIR,—In reply to your four inquiries as to my views on the effect of the National Insurance Bill upon voluntary hospitals, I have no hesitation in saying that:

(*a*) I believe that the voluntary hospitals will be so seriously affected that in two or three years they will have to close their doors for want of money to carry on or be taken over by the State. As Mr. Lloyd George has truly said, the first of these alternatives is "unthinkable," the second must be the result.

(*b*) I cannot see how anyone can possibly say at present what proportion of beds may or may not be maintained under the operation of the Bill. My hospital always is and will be full up, Insurance Bill or no Insurance Bill. For a long time back there has been a patients' waiting list of over 600. The factors are not yet all available. I refer to my preceding answer for the result of two or three years' further working.

(*c*) While the treatment of insured persons in voluntary hospitals ought certainly to be paid for, I think it will be impossible in practice to refuse admission of persons requiring hospital treatment on the ground of their being insured. A hospital so refusing would probably lose all its remaining subscribers.

(*d*) I do not think that the working classes, insured or not, can or ought to be shut out of the voluntary hospitals because Mr. Lloyd George and his advisers have not fully thought out their problems. The point of legal right cannot be pressed to the exclusion of the sick or injured in order to prove to Mr. Lloyd George how wrong he is. The voluntary hospitals must go on with their work as before until compelled by lack of funds to close their doors.

I give these answers in the light of the practice and position of my own hospital, Glasgow Royal Infirmary, which is somewhat exceptionally placed at present because of our being engaged in the great work of reconstructing the whole hospital at a cost of about £500,000 as a Diamond Jubilee memorial of Queen Victoria. In order to complete the whole scheme with the necessary accessory buildings of a great general hospital and to build a much-needed new dispensary we still want about £70,000. In any case the raising of so much additional money would have been a serious matter. I ask myself: Will it be possible with the Insurance Bill in operation?

Thanking you for the opportunity of expressing my views.—I am, yours truly,

J. D. HEDDERWICK.

Glasgow Royal Infirmary,
Glasgow, December 4, 1911.

